# Comparative Protein Composition Analysis of Goat Milk Produced by the Alpine and Saanen Breeds in Northeastern Brazil and Related Antibacterial Activities

**DOI:** 10.1371/journal.pone.0093361

**Published:** 2014-03-27

**Authors:** Whyara Karoline Almeida da Costa, Evandro Leite de Souza, Edvaldo Mesquita Beltrão-Filho, Gracy Kelly Vieira Vasconcelos, Tatiane Santi-Gadelha, Carlos Alberto de Almeida Gadelha, Octavio Luiz Franco, Rita de Cássia Ramos do Egypto Queiroga, Marciane Magnani

**Affiliations:** 1 Department of Food Engineering, Technology Center, Federal University of Paraíba, João Pessoa, Paraíba, Brazil; 2 Department of Nutrition, Health Sciences Center, Federal University of Paraíba, João Pessoa, Paraíba, Brazil; 3 Department of Cellular Biology, Exact and Nature Science Center, Federal University of Paraíba, João Pessoa, Paraíba, Brazil; 4 Center of Biochemical and Proteomic Analyses, Pos-Graduation of Biotechnology and Genomic Sciences, Catholic University of Brasilia, Distrito Federal, Brazil; Aligarh Muslim University, India

## Abstract

The protein composition of goat milk differs between goat breeds and could present regional trends. The aim of this study was to comparatively analyze the protein composition of goat milk produced by the Alpine and Saanen breeds in northeastern Brazil and to evaluate the antibacterial activity of its protein fractions. SDS-PAGE, 2-DE electrophoresis and RP-HPLC analyses revealed the absence of αs1-casein in the milk of both breeds and no differences between the αs2-casein, β-casein, β-lactoglobulin and α-lactalbumin profiles. The amounts of soluble proteins and β-casein hydrolysis residues were higher in Saanen milk. Only the protein fraction containing the largest amounts of casein (F_60–90%_) inhibited bacterial growth, with MIC values between 50 and 100 mg/mL. This study describe for the first time three important points about the goat milk protein of two Brazilian goat breeders: absence of α-s1 casein in the protein profile, differences between the milk protein composition produced by goats of Alpine and Saanen breeders and antibacterial activity of unbroken proteins (casein-rich fraction) present in these milk.

## Introduction

Milk is characterized as a food matrix of simple access and is abundant in nutrients, such as carbohydrates, proteins, minerals, and vitamins [1]. The use of non-bovine milk as an alternative protein source has increased lately, as hypersensitivity to cow's milk proteins remains one of the major causes of food allergies [2]. Thus, the crude composition of the protein fraction of milk from different species of mammals has been characterized, and among them, goat milk stands out due to the presence of compounds with important metabolic properties for human nutrition [3].

The protein fractions of bovine and goat milk are qualitatively very similar, and the major difference among these milks is related to the proportions and classes of caseins [4]. The hypoallergenicity of goat milk compared to bovine milk relates to the absence or low levels of αs1-casein (αs1-cn) in goat milk, and this fraction has been regarded as having allergenic potential, as determined by specific haplotypes [5]. Studies based on molecular techniques have suggested that goat alleles present in different breeds result in significant differences in milk casein fraction, and these alleles can exhibit regional trends for these characteristics [6], [7].

Goat milk proteins are more digestible than those found in bovine milk [8], and the protein fraction of goat milk has higher levels of six out from the ten essential amino acids present, when compared to bovine milk [3]. In addition, the unique composition of goat milk, combined with its nutritional value, is related to the release of protein fragments during digestion or technological processing, which are able to perform specific biological activities [9]. Studies involving milks from various animal species, including goats, indicate that milk and whey proteins, as well as the peptides generated from these proteins, have important biological activities, such as antimicrobial, immunomodulatory, antioxidant, antithrombotic, hypocholesterolemic and antihypertensive activities [10], [11], [12].

The interspecies differences can be evaluated using proteomic analysis; however, studies evaluating the nitrogen profile and protein fractions of milk from different breeds of the same species, particularly among goat breeds, are still scarce [13], [14], [15]. In this context, studies with an emphasis on the comparative proteomic evaluation of goat milks are important to identify their protein fraction; these studies will help characterize the alternative hypoallergenic protein sources or protein sources that have important technological properties for processing, leading to the preparation of products with added value. Additionally, characterization of the milk proteins can help guide genetic improvements in the goat herds, thus increasing its use in human nutrition [14], [15]. Milk proteins could also be important sources of antimicrobial peptides, natural agents with potential application as biopreservatives to control the growth and survival of bacterial pathogens in food matrices [16]. It has been found that proteins present in the milk and whey of caprine and bovine breeds are precursors of bioactive components, by contributing to particular antimicrobial activities against a broad spectra of pathogenic and spoiling bacteria [17], [18], [19].

Considering these aspects, the aim of this study was to comparatively analyze the protein composition of milk produced by Alpine and Saanen goats in northeastern Brazil and to evaluate the antimicrobial activity of their protein fractions against some strains of pathogenic bacteria.

## Materials and Methods

### Goat milks

From the homogeneous herds of the Goat Sector, Center for Social and Agricultural Sciences (Federal University of Paraíba, Bananeiras, Brazil), Alpine (n  =  10) and Saanen (n  =  10) goats with similar weights at 30±5 days of lactation were selected. No permits were required for the described study, which complied with all relevant regulations, since only milk was collected and none sacrifice was necessary. The test duration corresponded to the initial lactation stage. Goats of both breeds (Alpine and Saanen) received the same diet, consisting of complete feed (concentrate, Tifton hay and forage palm) in a bulky/concentrate feed management. For each breed, the total produced milk in one day was collected and mixed to obtain a pool; from each pool a total of 10 milk samples were analyzed in triplicate and all analyses were performed in three independent occasions (replicates).

Milk was obtained under proper hygienic-sanitary practices during the milking sessions, which were performed at 6:00 a.m. and 3:00 p.m. on the same day. Milk samples were composed of aliquots proportional to the yield in each milking shift and kept under cooling temperature (±10°C) until further analysis in a maximum interval time of 1 h. The average physiochemical values of the milks used in this study, determined in accordance with the procedures described by AOAC [20], are shown in Table 1. For the microbiological evaluation of the milk, counts of total and thermotolerant coliforms, mesophilic bacteria and coagulase-positive *Staphylococcus* and analysis of the presence of *Salmonella* spp. and *Listeria monocytogenes* were performed according to the procedures described by APHA [21]. All milk samples assessed in this study showed a satisfactory microbiological quality, according to the current Brazilian legislation [22].

**Table 1:** Mean values (± standard deviation) of the physicochemical parameters of goat milk produced by the Alpine and Saanen breeds in Northeastern Brazil.

Variables	Alpine	Saanen
Total Proteins (g/100 g)	3.60 (± 0.07)	3.15 (±0.01)
Lactose (g/100 g)	5.02 (±0.01)	4.85 (±0.01)
Fat (g/100 g)	3.45 (±0.07)	3.55 (±0.21)
Humidity (g/100 g)	90.93 (±1.19)	88.39 (±0.08)
MR^a^ (g/100 g)	0.61 (±0.06)	0.68 (±0.05)
Density (g/cm^3^)	1.028 (±0.08)	1.030 (±0.08)
Acidity^b^ (g/100 g)	0.17 (±0.02)	0.17 (±0.01)

a Mineral residue; ^b^ Acidity in lactic acid.

### Obtaining the crude protein extract and protein fractions

Milk samples were subjected to centrifugation (3000 g for 20 min at 4°C) to separate the lipid phase. The skim milk was dialyzed against saline solution (0.85 g/100 mL) for 24 h, with water changes every 2 h, to obtain the crude protein extract (CPE). From the skim milk, the protein fractions were separated through precipitation with ammonium sulfate [(NH_4_)_2_SO_4_] and isoelectric precipitation (casein concentrate). The precipitation with ammonium sulfate (Merck, Brazil) was performed at saturation intervals (w/v) of 0–30%, 30–60%, 60–90% and 90–100% (F_0−30%_, F_30−60%_, F_60−90%_ and F_90−100%_) [23], and the isoelectric precipitation was conducted at pH 4.6 with 1.0 M HCl (Merck, Brazil). The casein concentrate was washed three times by centrifugation (5000 g for 20 min at 4°C) using distilled water and toluene (0.05%, v/v) solubilized at pH 7.0 with 1.0 M NaOH (Merck, Brazil). The precipitation-washing-dissolution cycle was performed twice [24]. After dialysis against deionized water, all precipitated fractions and the CPE were frozen (–20°C), freeze-dried under a vacuum (–80°C) and stored at 18±2°C until analysis. The content of the soluble proteins in the CPE and the precipitated fractions were determined using the Bradford assay [25].

### Chromatography using an RP-HPLC system

The casein concentrates obtained by isoelectric precipitation were subjected to reversed phase-HPLC (RP-HPLC), as described by Jaubert and Martin [26]. The samples were analyzed on a C4 Vydac 214 TP 5415 column in an HPLC system with a Waters 600E pump, a UV/Vis diode array helium degasser detector, and a Rheodyne injector that used the Millenium software v. 3:05:01. Solvent A consisted of 1.06 mL/L TFA in ultrapure water, and solvent B consisted of 1 mL TFA, 800 mL acetonitrile, and 200 mL ultrapure water. The flow rate was maintained at 1 mL/min; analyses were performed at 40°C, and the eluent was monitored at 214 nm. Within 54 min, a linear gradient of 350–620 mL/L Solvent B was applied [26], [27]. The samples were prepared using 0.5 mg casein concentrate dissolved in 1 mL of buffer (100 mM Tris-HCl, 8 M urea, 13 g/L trisodium citrate, 20 mM dithiothreitol) at pH 7.0. The material was kept for 1 h at 37°C, added of 10 mL Solvent A with urea (6 M). The pH was adjusted to 2.1 to 2.2 through the addition of 0.5 mL TFA solution (100 mL/L).

### Gradient SDS-PAGE electrophoresis

SDS-PAGE assays were performed using a concentration gradient from 7.5% to 20% in the presence of β-mercaptoethanol, according to the method described by Laemmli [28]. The lyophilized samples (CPEs, fractions, and casein concentrates) were dissolved to a final protein concentration of 2 mg/mL in a buffer consisting of 0.0625 M Tris (pH 6.8) containing 2% SDS, 10% glycerol, 5% β-mercaptoethanol, and 10 μL bromophenol blue (0.0002%). The samples were then heated in an oven at 100°C for 10 min and centrifuged (5000 g for 5 min at 18±2°C). An aliquot of 10 μL supernatant was applied to the gradient gel. SDS-PAGE was performed under constant amperage (30 mA), and at the end, the gels were fixed with 10% trichloroacetic acid (TCA) and stained with 0.005% Coomassie Brilliant Blue solution R-250 for 3 h. The excess dye was removed using a destaining solution containing 5% methanol and 7% acetic acid in deionized distilled water. The gels were scanned on an Image Scanner III (GE Healthcare Life Science) using Labscan Software 6.0 and were subsequently analyzed to verify the molecular weights of the constituent proteins of each sample.

### Two-dimensional electrophoresis (2-DE)

Two-dimensional electrophoresis was performed according to protocols published by O'Farrell and Klose [29] and Wang et al. [30]. For this, 0.3 mg of each CPE were weighed, diluted in 2.5 mL rehydration buffer solution containing 8 M urea, IPG buffer 4 – 7, dithiothreitol (DTT), 2% CHAPS (3-[(3-cholamidopropyl) dimethylammonio]-1-propanesulfonate), and 0.002% bromophenol blue. The samples were applied to linear-type 13 cm strips (GE Healthcare Life Science), pH 4 to 7, allocated in the IPG Box with mineral oil on the surface, and kept overnight (15 – 20 h) at room temperature (18±2°C).

Subsequently, the strips were submitted to isoelectric focusing using the following conditions: 1 h at 100 V, 1 h at 300 V, 1 h at 500 V, 1 h at 1000 V, gradient to 4000V in 1 h, gradient to 8000 V in 1 h, and finally 10 h at 8000 V, with subsequent balancing in two steps of 15 min using rehydration solution with DTT and rehydration solution with iodoacetamide, respectively. The balanced strips were immediately placed on the SDS-PAGE gel to run the second dimension and were indexed in ascending order from left to right (4 → 7); the gel was prepared with 15% polyacrylamide in the presence of SDS. Filter paper soaked in 3 μL of the molecular mass marker was placed, and then, the plate was sealed with 0.5% agarose. The run was kept under constant amperage (30 mA), and the gels were fixed with 10% TCA and stained with 2% Coomassie Brilliant Blue solution G-250. The excess dye was removed using a destaining solution containing 5% methanol and 7% acetic acid in deionized distilled water. To check the repeatability of 2-DE electrophoresis, regarding the position of the spots, all analyses were performed in three different occasions and showing R2 higher than 0.95. The gels were scanned on Image Scanner III (GE Healthcare Life Science) using Labscan software 6.0 and were subsequently carefully analyzed using Image Master 2D Platinum 7.0 (GE) software to verify the isoelectric points and molecular weights of the samples. The scatter plots built based on detection and matching of the protein spots were used to calculate the slopes values and the correlation coefficient (R2).

### Assessing the antibacterial activity of fractions precipitated with ammonium sulfate

For the antibacterial activity assays, freeze-dried samples of F_0−30%_, F_30−60%_, F_60−90%_, and F_90−100%_ were solubilized (1 mg/mL) in PBS buffer (pH 7.6), under mild stirring conditions. *Bacillus subtilis* CCT 0516, *Escherichia coli* ATCC 2536, *Pseudomonas aeruginosa* ATCC 23243 and ATCC 8027, and *Staphylococcus aureus* ATCC 25619 and ATCC 25925 were used as test strains, regarding their importance as food-related pathogens. The inocula of the bacterial strains were prepared in suspensions of sterile saline solution (0.85% NaCl w/v) from overnight cultures grown on nutrient agar at 37°C. The suspensions were standardized according to the turbidity of the 0.5 tube in the McFarland scale, which corresponds to a concentration of approximately 10^8^ colony forming units per mL (CFU/mL). Then, serial dilutions were prepared (1∶9 v/v) in 0.85% sterile saline to obtain the desired inoculum (approximately 10^6^ CFU/mL). The antibacterial activity of the fractions was evaluated in six concentrations (100, 50, 25, 12.5, 6.25, 3.125 mg/mL) using the 96-well plate microdilution method. Aliquots of 100 μL of LB broth were added to the wells and mixed with 50 μL of each fraction in serial dilution. Wells were then inoculated with 50 μL of the bacterial suspension or received the same volume of sterile saline as a negative control. Positive controls were obtained using LB (100 μL) added of the bacterial suspensions (50 μL) and PBS (50 μL) [31], [16]. The plates were incubated at 37°C for 24 h and read at 630 nm. The lowest concentration (highest dilution) of each fraction that showed no bacterial growth was regarded as the minimal inhibitory concentration (MIC). The verification of the bacteriostatic or bactericidal activity was performed by the inoculation of the system that showed no growth in the culture medium without the presence of the test compounds. The activity of the samples was considered bacteriostatic when growth was observed following re-inoculation, and the sample was considered bactericidal in the absence of microbial growth [32].

## Results and Discussion

**Figure 1:**
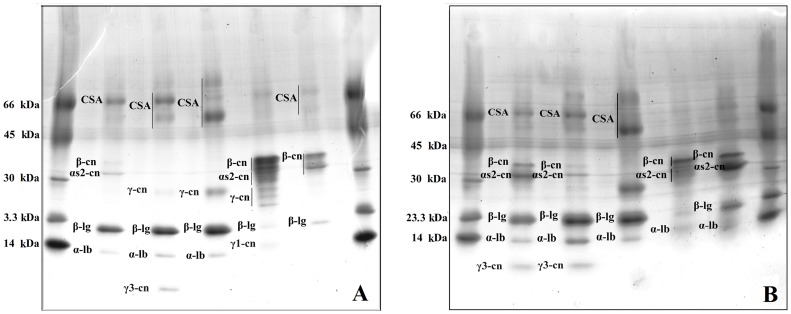
Electrophoretic analyses of goat milk proteins produced by the Alpine and Saanen breeds in Northeastern Brazil precipitated with ammonium sulfate, analyzed by gradient (7 to 20%) SDS-PAGE. Image A: Alpine breed. (M) Molecular markers*, (A) Crude protein extract, (B) Fraction F_0−30%_, (C) Fraction F_30−60%_, (D) Fraction F_60−90%_, and (E) Fraction F_90−100%_. Image B: Saanen breed. (M) Molecular Markers*, (A) Crude protein extract (B) Fraction F_0−30%_, (C) Fraction F_30−60%_, (D) Fraction F_60−90%_, and (E) Fraction F_90−100%_. * BSA (66.0 kDa), egg albumin (45.0 kDa), carbonic anhydrase (30.0 kDa), trypsin inhibitor (23.3 kDa) and egg lysozyme (14.0 kDa).

**Figure 2:**
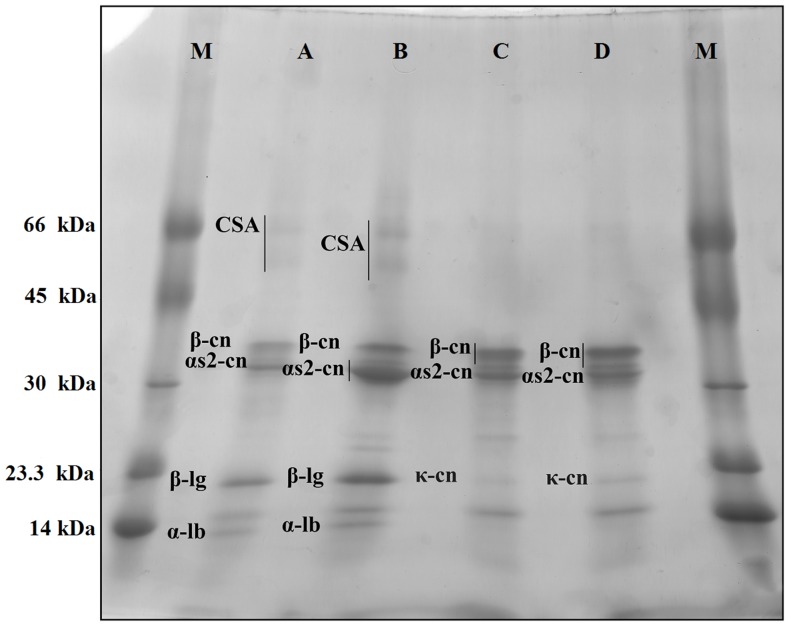
Gradient SDS-PAGE (7 to 20%) analyses of goat milk produced by the Alpine and Saanen breeds in Northeastern Brazil. (M) Molecular markers*, (A) Alpine crude protein extract, (B) Saanen crude protein extract, (C) casein concentrate from Alpine milk, and (D) casein concentrate from Saanen milk. * BSA (66.0 kDa), egg albumin (45.0 kDa), carbonic anhydrase (30.0 kDa), trypsin inhibitor (23.3 kDa) and egg lysozyme (14 kDa).

The SDS-PAGE analysis of the CPE, casein concentrate and fractions of Alpine and Saanen goat milk precipitated with ammonium sulfate revealed no band indicative of αs1-cn (Figures 1 and 2). Milk analyzed using SDS-PAGE shows unique patterns that allow for the identification of variations between the different species or breeds, as the main bands correspond to αs1-cn and β-casein (β-cn) [3], [15].

Studies involving different goat breeds, including the French-derived Saanen and Alpine breeds, have demonstrated that the amount of αs1-cn in goat milk depends on the type of allele of the αs1-cn locus that is being expressed. The alleles A and B are designated as strong alleles, resulting in the greatest amount of αs1-casein in goat milk, whereas the E allele produces intermediate amounts and the weak allele F produces the least concentrations of αs1-casein in goat milk. For the breeds analyzed in this study, the expression of the intermediate (E) and weak (F) alleles for αs1-cn have been reported, which correspond to the presence of intermediate and low amounts of αs1-cn, respectively, in the milk produced. Milk samples produced by Saanen goats commonly have low amounts of αs1-cn when compared to the amount found in milk produced by Alpine goats [14], [26], [33]. The absence of αs1-cn in the goat milk, as observed in the milk samples evaluated in this study, corresponds to the non-expression of the E or F alleles, or even to the genotype of the null alleles O_1_, O_2_, and N [14]. The frequency of the alleles of the αs1-cn locus has shown to have marked differences between goat breeds and regional trends, with the detection of strong alleles in the Mediterranean area, intermediate alleles in Africa, France and Spain, and null alleles in Switzerland. However, a wide variation in the distribution of these alleles has been reported worldwide for the Saanen and Alpine breeds [33].

Regarding the absence of αs1-cn in the milk from both goat breeds, it is possible that the environmental conditions in Northeastern Brazil, which is characterized as a region of semi-arid climate with alternating rainy and dry seasons [34], may influence the (non) expression of the alleles of the αs1-cn locus. It is noteworthy that the analyzed milks are hypoallergenic protein sources; the absence of αs1-cn has been recognized as one of the characteristics for the low allergenicity exhibited by goat milk [35], [36]. In this context, goat milk has been considered a high-quality raw material for use in the manufacture of food products for children and the elderly, as well as for certain groups with particular nutritional requirements [8].

The content of the soluble proteins was 19.6±8.9 and 46.7±6.2 mg/mL in CPE obtained from Alpine and Saanen goats, respectively, and these results are in agreement with the electrophoretic profiles from the SDS-PAGE analysis, which showed more intense bands for the CPE obtained from the Saanen milk (Figures 1 and 2). The composition of the protein fraction strongly influences some technological properties of the products. The low content or absence of αs1-cn in the milk samples in relation to other caseins makes it less attractive for processing into derived dairy products. However, the protein content affects the milk coagulation rate as well as the processing yield, taste, and consistency of the derived products [8], [14], [37]. Although absence of αs1-cn was observed in both types of milk tested, the detected difference in the amount of soluble proteins (P ≤ 0.05) suggests that the processing of the milk produced by Saanen goats would have a higher yield than the milk from the Alpine goats for animals submitted to a feedlot management system, as used in this study. Similar results were reported by Moatsou et al. [33], who evaluated the relative amounts of the major caseins in the total casein fraction of milk from four different goat breeds, including Alpine and Saanen.

Similar to what has been observed for the αs1-cn content, the β-cn, and αs2-casein (αs2-cn) profiles of the milk samples were similar in the SDS-PAGE analyses, with bands of molecular weight approximately 36 kDa and 32 kDa, respectively. However, the milk from the Saanen goats showed a higher number of bands, which is indicative of casein hydrolysis; these bands in the electrophoretic profile corresponded to a band of 10 kDa, which was identified as γ3-casein (γ3-cn), and to lightly stained bands in the region 30 kDa and 23.3 kDa (Figures 1 and 2). The plasmin system present in goat milk contributes to the generation of polypeptide fragments, particularly from the hydrolysis of β-cn. These fragments can be identified in SDS-PAGE as bands of low molecular weight (≤ 30.0 kDa) [38], [39], suggesting that the substrate for this hydrolysis is present in higher amounts in milk from Saanen goats.

SDS-PAGE also showed the presence of whey proteins, which could be best observed in the profiles generated from the CPE of both milks studied, when compared to the profiles of the caseins concentrates (Fig. 2). Whey proteins with lower molecular weight may interact with caseins, particularly with the β-cn fraction. In the present study, this interaction or overlap of whey proteins and β-cn could be viewed as an increase in the intensity of the band assigned to β-cn, which was also reported in a previous study of milk from Garnica goats [40]. There were no differences in the profiles of the whey proteins from the milks of Saanen and Alpine goats. The whey proteins included albumin (caprine serum albumin -CSA), β-lactoglobulin (β-lg), and α-lactalbumin (α-lb) in the CPE and were identified as bands of approximately 66.0 kDa, 20.0 kDa, and 14.0 kDa, respectively (Figures 1 and 2). The similarity in the profiles of the Saanen and Alpine CSA and α-lb proteins agrees with results of previous studies involving milks from various goat breeds [41], [42]. However, there are reports that β-lg, the main whey protein found in the milk of ruminants, has a high degree of genetic polymorphism in goats, especially in Sicilian breeds [43].

**Figure 3:**
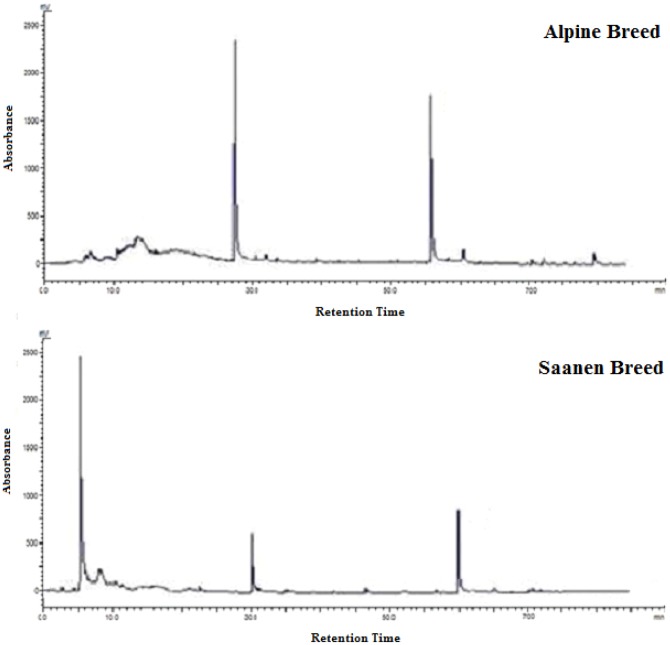
Chromatograms of casein concentrates of goat milk produced by the Alpine and Saanen breeds in Northeastern Brazil using RP-HPLC. Image A: Casein concentrate from Alpine breed. Image B: Casein concentrate from Saanen breed. (1) κ-casein, (2) αs2-casein, and (3) β-casein.

The soluble protein content found in each fraction precipitated with ammonium sulfate varied among the milks. The F_0−30%_ was 181.61±0.01 and 132.21±0.01 mg/mL and the F_30−60%_ was 100.39±31.09 and 198.49±0.01 mg/mL for the Saanen and Alpine milk, respectively. The F_60−90%_ was 29.47±34.74 and 35.94±17.44 mg/mL and the F_90−100%_ was 27.61±13.03 and 14.12±59.71 mg/mL for the Saanen and Alpine milk, respectively. The protein surface hydrophobicity decreases with increasing protein content as a result of the reduction in the distance between collision proteins [44]. Thus, in the F_0−30%_ and F3_0−60%_, the proteins with lower hydrophobicity are precipitated, while in the F_60−90%_, the largest amounts of goat milk caseins are precipitated; these proteins are relatively hydrophobic molecules that are randomly structured with relatively lower secondary and tertiary structures, and the hydrophobic residues are quite exposed on the micelle surface [45]. Thus, we can say that the milk from Saanen goats has higher amounts of casein when compared to the milk from Alpine goats; this can also be observed by comparing the intensity of the bands related to the caseins in the electrophoretic profiles (Fig. 1) and in the graphics generated by the RP-HPLC analysis for the casein concentrates of the goat milks studied (Fig. 3).

As for the isoelectric precipitation of casein from the milks, the electrophoretogram (Fig. 2) revealed similar patterns for β-cn, αs2-cn, and κ-casein (κ-cn) between the goat milks studied; these proteins were identified as bands of approximately 36 kDa, 32 kDa, and 22 kDa, respectively. According to the results of the SDS-PAGE analysis of the CPE, the band corresponding to αs1-cn was not identified, while a difference was observed in the protein migration of fragments from the hydrolysis of β-cn by the plasmin system; these fragments are present in greater amounts in the milk from Saanen goats. In the present study, no differences were observed for αs2-cn between the milks, although at least three distinct genetic variants for this casein are recognized in goats [40]. These findings support the potential of adaptive capacity shown by goats [46], which may be an influential factor in establishing the similarity between the casein profiles of milk from the Saanen and Alpine goats used in this study.

The electrophoretic pattern of the αs-cn in goat milk is presented as a complex of three bands with similar intensity, approximately weighing between 45 and 30 kDa [40]; this banding pattern was not identified in this study due to the absence of αs1-cn (Fig. 1 and 2). However, the electrophoretic representation of αs-caseins in goat milk has very subtle differences in the migration between bands, which have very similar molecular weights and can overlap, hindering the precise distinction between the types of αs-caseins present in goat milk [24]. To obtain a reliable casein profile, the casein concentrates of the milk from Alpine and Saanen goats were assessed using RP-HPLC and the peak corresponding to αs1-cn was not observed in chromatograms of the both breeds (Fig. 3), according patterns previously published of the milk from the same goat breeds [13], [33].

**Figure 4:**
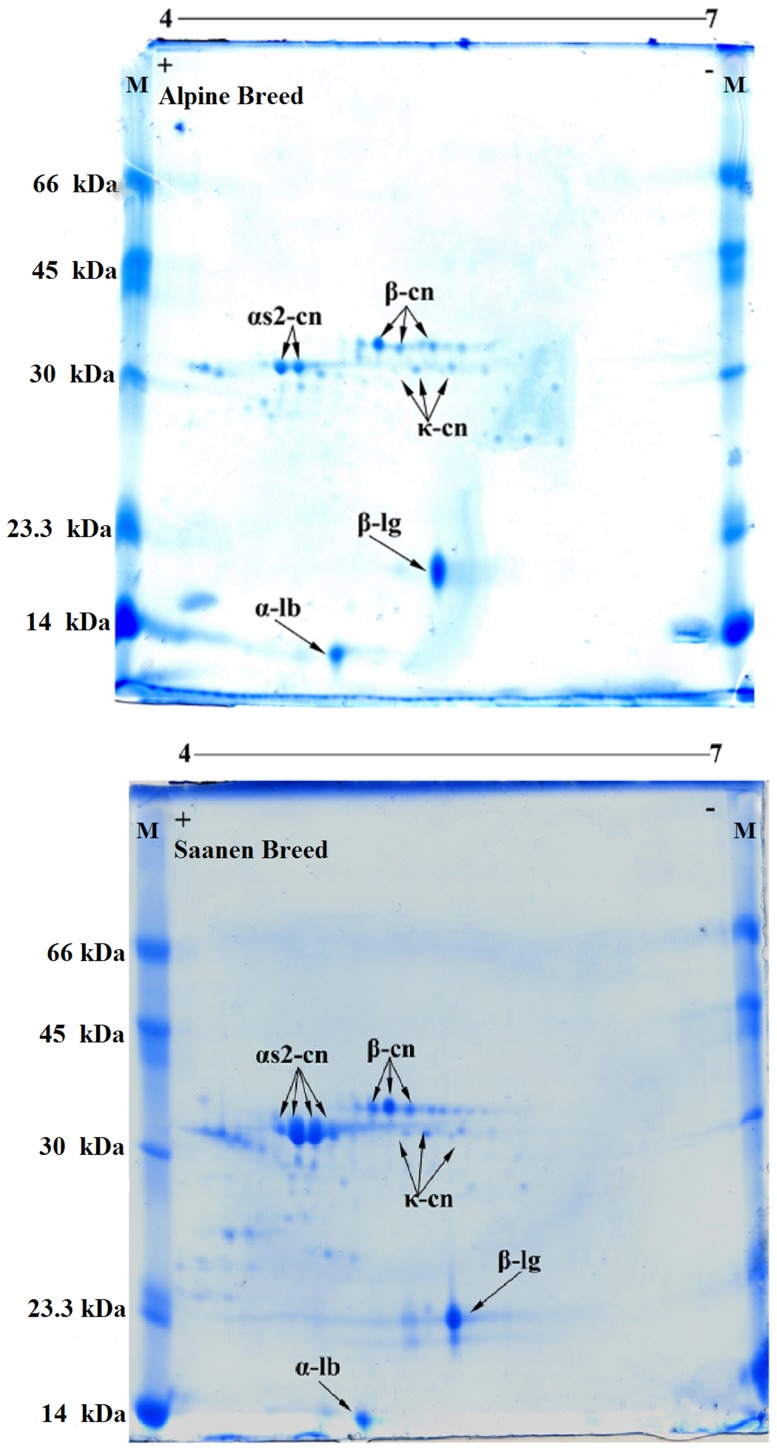
Comparison of two-dimensional electrophoresis of goat crude protein extract milk produced by the Alpine (AM) and Saanen (SM) breeds in Northeastern Brazil. The vertical direction shows the molecular mass in kDa, and the horizontal direction shows the isoelectric focusing at pH 4–7. A: Molecular markers: BSA (66.0 kDa), egg albumin (45.0 kDa), carbonic anhydrase (30.0 kDa), trypsin inhibitor (23.3 kDa) and egg lysozyme (14.0 kDa). Gels were Coomassi stained.

In the analysis of proteins present in the CPE of milk from Saanen and Alpine goats using 2-DE, αs2-cn (32 kDa and IP ≈ 4.7), β-cn (36 kDa and IP ≈ 5.2), and κ-cn (31 kDa and IP ≈ 5.4) as well as the whey proteins β-lg (20 kDa and IP ≈ 5.6) and α-lb (14 kDa and IP ≈ 5.0) were identified, reinforcing the findings of the one-dimensional SDS-PAGE (Fig. 4). The differences in protein concentration between the milks studied could be observed in the distinct number of spots that were detected, which was higher in the milk from Saanen goats. The products obtained from the hydrolysis of β-cn observed in SDS-PAGE of each CPE and in the casein concentrates in both milks studied were also visualized using the 2-DE gel (Fig. 4), and these degradation products are generally identified as polypeptides [15].

The results of the antibacterial activity assays, which were performed with all fractions precipitated with ammonium sulfate, showed that only the F_60:90%_ fractions obtained from the milk of either Alpine or Saanen goats were capable of inhibiting the bacterial strains tested, with MIC values ranging from 50 to 100 mg/mL. The lowest MIC value (50 mg/mL) was observed against *B. subtilis* CCT 0516, while the MIC was 100 mg/mL against all other strains tested (*E. coli* ATCC 2536, *P. aeruginosa* ATCC 23243, *P. aeruginosa* ATCC 8027, *S. aureus* ATCC 25619 and *S. aureus* ATCC 25925). The F_60−90%_ fraction of the both milks evaluated showed inhibitory activity of a bacteriostatic nature against all bacterial strains tested.

Although there have been no studies on the antibacterial activity of the protein fractions obtained from goat milks, it is known that proteins from bovine, which are naturally present in milk, such as lactoferrin and lactoferricin, may exhibit the ability to inhibit microorganisms [11], [12]. Furthermore, some bovine and caprine whey protein-derived peptides have been reported as having significant antimicrobial activity against some bacteria tested in the present study, such as *E. coli*, *L. monocytogenes* and *S. aureus* [12], [16], [31]. A previous study found no inhibitory effect on the growth of *L. monocytogenes* when the intact goat whey protein was tested [19]. The authors suggested that the absence of inhibitory effect probably occurred because milk proteins have a latent physiological activity encoded in their primary structure, which becomes more active when the protein is cleaved during digestion or fermentation.

Studies performed with peptides generated from bovine milk α-2 casein, belonged to the positively charged C-terminal region, showed activity against a wide variety of Gram-positive and Gram-negative bacteria with MIC values ranging from 21 to 168 mg/mL and 10.7 to 171.2 mg/mL, respectively [47]. The MIC values (50 and 100 mg/mL) observed for the fraction F_60−90%_ of the goat milk included in this study are in accordance with the MIC values range reported in previous studies involving bovine and caprine milk peptides. However, in the present study, even the intact goat proteins, specially α-2 caseins which is the main protein present in this fraction (considering the absence of the α-1 casein in milk analyzed), showed activity against the tested bacterial strains, suggesting that the reduction of the unbroken chain in the C-terminal region of the intact protein could enhance the antimicrobial activity of the goat milk caseins tested.

## Conclusions

Milks produced by Alpine and Saanen goats in Northeastern Brazil do not have αs1-casein in their casein composition and do not differ from each other, with respect to casein composition. These breeds have the potential to be interesting hypoallergenic protein sources. The milk from Saanen goats has higher amounts of casein when compared to the milk from Alpine goats. In addition, the inhibitory effect of the F_60−90%_ fraction in milk from both breeds against the pathogenic bacteria tested suggests that the casein found in milk from Alpine and Saanen goats can be a source of bioactive peptides. However, further studies are necessary to subsidize their use more efficiently.
